# Energy devices safety and impact on video-assisted thoracoscopic lung lobectomy postoperative course: monopolar electrocautery versus ultrasonic dissector

**DOI:** 10.1186/s13019-021-01421-1

**Published:** 2021-03-20

**Authors:** Maria Cattoni, Nicola Rotolo, Elisa Nardecchia, Silvia De Maio, Lorenzo Dominioni, Andrea Imperatori

**Affiliations:** Center for Thoracic Surgery and Center for Minimally Invasive Surgery, Department of Medicine and Surgery, University of Insubria, Via Guicciardini 9, 21100 Varese, Italy

**Keywords:** VATS lobectomy, Energy device, Monopolar electrocautery, Ultrasonic dissector, Postoperative outcomes, Non-small cell lung cancer

## Abstract

**Background:**

This study aims to compare safety and impact of monopolar electrocautery and ultrasonic dissector (Harmonic ACE Plus®) on postoperative short-term outcomes after video-assisted thoracoscopic (VATS) lobectomy and lymphadenectomy for lung cancer.

**Methods:**

We analyzed the prospectively collected data of 140 consecutive patients [59% male; median age: 71(IQR:62–76) years] undergoing VATS lobectomy and lymphadenectomy in our institution between October 2016 and November 2019. Patients were divided in two groups based on device used: monopolar electric hook in 79 cases (Group A); ultrasonic dissector in 61(Group B). Energy instrument-related intraoperative accidents, hemothorax/chylothorax incidence, total pleural effusion volume at 48 postoperative hours and chest tube duration were compared between groups. Multivariable analysis was performed to test energy device as possible independent risk factor either for increased pleural effusion volume or for prolonged chest tube duration.

**Results:**

No intraoperative accidents due to energy device occurred. No hemothorax was recorded. Postoperative chylothorax incidence was slightly higher in Group A (2.5% vs 0%; *p*-value = 0.21). Total pleural effusion volume at 48 h was significantly higher in Group B: 400 (285–500) vs 255 (150–459) ml (*p*-value = 0.005). Chest tube duration was similar in the two groups: 5 (3–9) vs 5 (3–8) days (*p*-value = 0.77). At multivariable analysis the energy device used was not associated with increased pleural effusion volume (*p*-value = 0.43) nor with prolonged chest tube duration (*p*-value = 0.28).

**Conclusions:**

Monopolar electrocautery and Harmonic ACE Plus® were safe and had a similar impact on short-term outcomes after VATS lobectomy and lymphadenectomy, suggesting that energy devices choice could be left to surgeon’s preference.

## Background

In thoracic surgery, the introduction over the last two decades of a variety of energy devices together with endoscopic staplers has allowed to perform major video-assisted thoracoscopic (VATS) procedures under safe conditions and has increased the number of surgical interventions performed by mini-invasive approach.

Focusing on VATS lung lobectomy and lymphadenectomy, energy tools such as electric hook (monopolar energy), Harmonic ACE Plus® (ultrasound energy), LigaSure™ (bipolar energy), Enseal® (bipolar energy) and Thunderbeat® (ultrasound energy+ bipolar energy) are commonly used during pleural adhesiolysis, hilar dissection, small vessel sealing and lymph node removal [1–10]. However, to date the available information is insufficient to establish the superiority of any of these devices in enhancing the postoperative course after this type of procedure [4–6]. To our best knowledge, no study has directly compared the effect of traditional monopolar electric tools and of advanced ultrasonic dissectors on patients’ course after VATS major lung resections.

This study aims to compare surgical monopolar electric hook vs Harmonic ACE Plus® in terms of safety and impact on short-term postoperative outcomes after VATS lobectomy and lymph node dissection for non-small cell lung cancer.

## Methods

We analyzed the prospectively collected data of consecutive patients who underwent VATS lung lobectomy and lymphadenectomy in our center from October 1st 2016 to November 30th 2019. We excluded: patients with benign disease or lung metastases; those requiring conversion to thoracotomy; those undergoing extended resection to chest wall, mediastinum or another lung lobe and those requiring the use of both monopolar electric hook and Harmonic ACE Plus® energy device.

This study was approved by our University Hospital Ethic Committee and individual patient consent was obtained for each case.

All lung lobectomies and lymph node dissections were performed using a standardized three-port anterior approach [11]. Three experienced surgeons who had completed the learning curve for VATS lobectomy and lymphadenectomy carried out all surgeries as first operator. A 28 French chest tube was left in place after surgery until no air-leak was observed and effusion quantity was ≤250 ml/day. Postoperatively all patients were managed according to our protocol: perioperative respiratory physiotherapy; epidural analgesia/paravertebral block in association with non-steroid analgesics; heparin antithrombotic prophylaxis; intravenous fluid restriction; early oral feeding; early urinary catheter removal, early ambulation.

For each patient we collected the following data: age, gender, body mass index (BMI), smoking habit, forced expiratory volume in 1 s (FEV_1_), comorbidities, type of lobectomy, pleural adhesiolysis, surgery duration, tumor size, tumor histology and pathological stage according to the 8th edition TNM staging system, number of dissected lymph nodes, total pleural effusion volume during the first 48 postoperative hours, postoperative chest tube stay, postoperative length of stay, 30-day postoperative complications and cost of instruments used during surgery (energy devices, staplers, cartridges, clips).

Patients were divided in two groups based on the device used for tissue dissection: the electric hook (Group A) or the Harmonic ACE Plus® (Group B). Use of either instrument was left up to surgeon’s preference after intraoperative case evaluation; surgeons did not routinely use the same type of energy device.

Primary outcome was to assess and compare the safety of electric hook and Harmonic ACE Plus® during the surgical procedure. Secondary endpoint was to compare the impact of these instruments on postoperative course, using as benchmarks: postoperative hemo/chylothorax incidence, pleural effusion volume during the first 48 postoperative hours and postoperative chest tube duration.

Univariable and multivariable analyses were performed in order to test the energy device as possible independent risk factor for increased pleural effusion volume during the first 48 postoperative hours and for prolonged postoperative chest tube duration. Factors analyzed were age, gender, BMI, cardiac comorbidities, pleural adhesiolysis, type of resection (upper/middle lobe lobectomy vs lower lobe lobectomy), tumor characteristics (size and histology), number of resected lymph nodes, energy device used (Harmonic ACE Plus® versus electric hook), surgery duration, postoperative persistent air-leak (> 5 days after surgery).

To limit the influence of possible confounding factors on final results, a propensity matched analysis was performed using the following parameters for matching: gender, site of resection, surgeon. Primary and secondary outcomes were analyzed and compared in the matched cohort.

Continuous data were reported as median with interquartile range (IQR) and compared using T-test for normally distributed data and Mann–Whitney U test for non-normally distributed data. Categorical and count data were presented as frequencies and percentages and compared using Chi-square test or Fisher’s exact test if any expected frequency was less than 5. Univariable and multivariable analyses were performed by binary logistic regression, using as dependent variable cut-off value its overall median value. Multivariable analysis included only those variables that were statistically significant in univariate analysis. A *p*-value < 0.05 was considered significant. Statistical analysis was performed using SPSS 24.0 software (IBM Corp, Armonk, NY, USA).

## Results

During the study period 206 patients underwent lung lobectomy in our center. Of these, 66 patients were excluded from study: 44 patients were approached by thoracotomy, 10 underwent surgery for inflammatory disease or lung metastases, 7 required conversion from VATS to thoracotomy (4, intraoperative bleeding unrelated to energy devices; 2, locally advanced disease; 1, lung failure to collapse) and in 5 cases surgeon used both devices.

Thus, 140 patients undergoing VATS lung lobectomy and lymphadenectomy for non-small cell lung cancer were left for statistical analysis. Tissue and lymph nodes dissection were performed by electric hook in 79 cases (Group A) and by Harmonic ACE Plus® in 61 (Group B).

Patients’ clinical, surgical and pathological data are listed and compared between Group A and Group B in Table 1.

**Table 1:** Patients’ characteristics: comparison between group A (monopolar electric hook) and group B (harmonic ACE plus®)

Patients’ characteristics	Group A (*n* = 79)	Group B (*n* = 61)	*p*-value
Age, median (IQR) years	69 (62–75)	72 (64–76)	0.56
**Male, n (%)**	**41 (52)**	**42 (69)**	**0.043**
BMI, median (IQR) kg/m^2^	25 (22–28)	26 (24–29)	0.58
Current or former smoker, n (%)	58 (73)	47 (77)	0.62
FEV_1_, median (IQR) % of predicted	99 (83–112)	106 (90–119)	0.16
COPD, n (%)	15 (19)	15 (25)	0.42
Cardiac comorbidity, n (%)	24 (30)	14 (23)	0.33
Diabetes, n (%)	11 (14)	11 (18)	0.51
Previous malignancy, n (%)	29 (37)	17 (28)	0.27
Pleural adhesiolysis, n (%)	21 (31)	19 (37)	0.51
**Lower lobe lobectomy, n (%)**	**22 (28)**	**31 (51)**	**0.005**
Number of excised lymph nodes, median (IQR)	8 (6–11)	9 (5–13)	0.20
Intraoperative blood loss ≥100 ml, n (%)	15 (23)	14 (33)	0.26
Tumor size, median (IQR) cm	1.8 (1.5–2.8)	1.9 (1.5–3.0)	0.76
Tumor histology, n (%)			0.76
Adenocarcinoma	52 (66)	45 (74)	
Squamous cell carcinoma	12 (15)	9 (15)	
Neuroendocrine tumor	12 (15)	5 (8)	
Other histologies	3 (4)	2 (3)	
Pathological stage, n (%)			0.80
I	60 (76)	49 (80)	
II	15 (19)	8 (13)	
III	4 (5)	4 (7)	

*BMI* body mass index, *COPD* chronic obstructive pulmonary disease, *FEV*_*1*_ forced expiratory volume in 1 s, *IQR* interquartile range

Focusing on our primary outcome, no intraoperative complications due to energy device were recorded in both groups.

Surgical procedure lasted longer in Group B than in Group A: 216 (IQR: 193–265) vs 180 (IQR: 163–221) minutes, respectively (*p*-value < 0.001). The costs of instruments used during surgery was significantly higher in Group B than in Group A [2063.60 (IQR: 1775,07-2352,13) vs 2390.49 (IQR: 2137,34-2636.93) €, respectively (*p*-value< 0.001)], with no difference in terms of number of staplers and cartridges used.

Overall 30-day mortality was 0.7%: 1 patient in Group A died of pulmonary embolism on postoperative day 15. However, the 30-day mortality difference between the two groups was not statistically different (Group A vs Group B: 1.3% vs 0%; *p*-value = 1.00).

Overall 30-day morbidity was 35% (49/140), with 5.7% (8/140) patients presenting more than one complication. During postoperative stay, 30/140 (21%) patients developed persistent air leak (> 5 days), 10 (7%) pneumonia, 11 (8%) atrial fibrillation, 2 (1%) chylothorax, 1 empyema (< 1%), 1 transient dysphonia (< 1%) and 1 abdominal aortic occlusion (< 1%). No difference was detected comparing 30-day postoperative morbidity between Group A and Group B (32% vs 39% respectively; *p*-value = 0.34). Notably, the incidence of postoperative prolonged air-leak was similar in the two groups (23% vs 25%, respectively, *p*-value = 0.42).

Regarding secondary outcomes benchmarks, no postoperative hemothorax was reported in both groups. Chylothorax incidence was slightly higher in Group A (2.5% vs 0% Group B; *p*-value = 0.21).

Pleural effusion volume during the first 48 postoperative hours was significantly lower in Group A than in Group B: 255 (IQR: 150–459) vs 400 (IQR: 285–500) ml, respectively; *p*-value = 0.005. However, chest tube duration was similar in the two groups: 5 (IQR: 3–9) vs 5 (IQR: 3–8) days, respectively; *p*-value = 0.77. Likewise, there was no difference in terms of postoperative length of stay between Group A and Group B: 7 (IQR: 5–10) vs 7 (IQR: 5–10) days; *p*-value = 0.61.

At multivariable analysis, the type of energy device was not independently associated with increased total pleural effusion volume at 48 postoperative hours (Table 2), nor with prolonged postoperative chest tube duration (Table 3).

**Table 2:** Risk factors for increased (> 320 ml) pleural effusion volume during the first 48 postoperative hours

Risk factors	Univariable analysis	Multivariable analysis		
	HR (95%CI)	*p*-value	HR (95%CI)	*p*-value
Age (continuous)	1.00 (0.97–1.04)	0.89	–	–
Gender (male vs female)	2.47 (1.22–4.99)	0.012	1.62 (0.74–3.57)	0.23
BMI (continuous)	0.98 (0.91–1.06)	0.62	–	–
Cardiac comorbidities (none vs yes)	1.72 (0.80–3.70)	0.16	–	–
Pleural adhesiolysis (none vs yes)	1.22 (0.57–2.61)	0.61	–	–
**Site of resection (upper/middle vs lower)**	**3.95 (1.90–8.25)**	**< 0.001**	**3.10 (1.42–6.78)**	**0.004**
Tumor size (continuous)	0.99 (0.77–1.28)	0.96	–	–
Tumor histology (adenocarcinoma vs others)	1.17 (0.57–2.42)	0.67	–	–
N° of resected lymph nodes (continuous)	1.04 (0.97–1.12)	0.23	–	–
Energy device (ultrasonic vs monopolar)	0.39 (0.20–0.79)	0.008	0.73 (0.33–1.61)	0.43
**Surgery duration (continuous)**	**1.01 (1.01–1.02)**	**< 0.001**	**1.01 (1.00–1.02)**	**0.024**
Postoperative air-leak> 5 days (none vs yes)	1.44 (0.64–3.25)	0.39	–	–

*BMI* body mass index, *CI* confidence interval, *HR* hazard ratio

**Table 3:** Risk factors for prolonged (> 4 days) postoperative chest tube duration

Risk factors	Univariable analysis	Multivariable analysis		
	HR (95%CI)	*p*-value	HR (95%CI)	*p*-value
Age (continuous)	1.04 (1.00–1.08)	0.033	1.03 (1.00–1.07)	0.07
**Gender (male vs female)**	**2.67 (1.33–5.34)**	**0.006**	**2.47 (1.22–5.01)**	**0.012**
BMI (continuous)	0.94 (0.87–1.02)	0.13	–	–
Cardiac comorbidities (none vs yes)	1.14 (0.54–2.41)	0.73	–	–
Pleural adhesiolysis (none vs yes)	1.84 (0.85–4.00)	0.12	–	–
Site of resection (upper/middle vs lower)	1.63 (0.82–3.26)	0.17	–	–
Tumor size (continuous)	1.14 (0.87–1.49)	0.35	–	–
Tumor histology (adenocarcinoma vs others)	1.80 (0.86–3.75)	0.12	–	–
N° of resected lymph nodes (continuous)	1.00 (0.93–1.07)	0.89	–	–
Energy device (ultrasonic vs monopolar)	1.03 (0.53–2.01)	0.93	1.52 (0.71–3.24)	0.28
Surgery duration (continuous)	1.01 (1.00–1.01)	0.039	1.00 (1.00–1.01)	0.22

*BMI* body mass index, *CI* confidence interval, *HR* hazard ratio

A total of 140 patients were eligible for propensity score matching analysis. The matched sample included 56 patients: 28 from Group A and 28 from Group B. No difference in terms of primary and secondary outcomes was detected in the matched cohort, as shown in Table 4.

**Table 4:** Energy devices safety and impact on postoperative course in the matched cohort of 56 patients

Outcomes measures	Group A (*n* = 28)	Group B (*n* = 28)	*p*-value
Intraoperative device related accident, n (%)	0 (0)	0 (0)	1.00
Surgery duration, median (IQR) minutes	199 (179–242)	214 (187–241)	0.73
Postoperative hemothorax, n (%)	0 (0)	0 (0)	1.00
Postoperative chylothorax, n (%)	1 (3)	0 (0)	1.00
Pleural effusion volume during the first 48 postoperative hours, median (IQR) ml	308 (170–501)	350 (268–485)	0.45
Chest tube duration, median (IQR) days	4 (3–7)	5 (3–8)	0.46
Postoperative length of stay, median (IQR) days	7 (6–9)	8 (6–10)	0.77

*IQR* interquartile range

## Discussion

We focused on comparing the traditional monopolar electric hook and the advanced ultrasonic Harmonic ACE Plus® dissector for VATS lung lobectomy and lymphadenectomy for non-small cell lung cancer, in terms of intraoperative safety and patients’ postoperative course.

In our cohort no intraoperative complication due to energy devices was recorded, confirming the rarity of these injuries (1–2 cases per 1000 surgical procedures) [12]. Thermal burn is the most common cause of instrument-related injury and death during surgery and it is correlated with the device lateral thermal spread [13–17]. Monopolar electrocautery is generally associated with greater heat spread when compared to the advanced energy devices [13–17]. However, in our cohort no clinically relevant lesions to nerves or vessels occurred during surgery either using the monopolar electrocautery or the ultrasonic dissector. This suggests that surgeon awareness of energy device technology, application and common injury patterns, and the use of protected-tip cautery, may minimize complications related to energy-based instruments application for VATS lobectomy and lymphadenectomy [14, 16].

Postoperatively, no statistically significant difference of chylothorax incidence, chest tube duration and length of stay was detected between electric hook and Harmonic ACE Plus®, despite the increased postoperative pleural drainage in the ultrasonic dissector group that was not confirmed in the matched cohort. Moreover, energy device was not recognized as an independent risk factor of increased postoperative pleural effusion volume, and of prolonged chest tube duration.

Lack of significant differences in postoperative chylothorax and hemothorax incidence between monopolar electrocautery and ultrasonic dissector are consistent with results reported in the literature. Martucci et al., in their prospective randomized study of 119 patients undergoing open lung lobectomy and lymphadenectomy observed a similar postoperative chylothorax rate using the conventional electrocautery or the Ligasure™ (1.61% vs 1.75%, *p*-value = 1.00) [10]. Likewise, Yoshida et al. in their retrospective cohort of 112 patients did not detect any significant difference in terms of chylothorax incidence when small vessels division and lymph node dissection were performed by manual ligature and conventional electrocautery or by Ligasure™ (1.8% vs 0%; *p*-value = 1.00) [4]. However, in our study and in that of Yoshida et al., chylothorax rate was slightly higher after using blunt instruments and electrocautery than with the vascular-sealing device. These results could be explained by the mechanical principles underlying the two techniques. In contrast to electrocautery sealing by heat coagulation only, the Harmonic ACE Plus® combines heating and instrument blades mechanical pressure, fusing vessels walls and producing a permanent seal for vessels up to 5 mm [3–8, 14, 16, 18, 19]. This sealing ability should prevent any oozing or lymphatic leak. However, the low incidence of chylothorax and the absence of statistically significant difference between the two groups suggest that careful use of electric hook can also lead to satisfying results in terms of lymphatic leakage control.

In our series the total amount of pleural effusion volume collected at 48 h after surgery was significantly higher after using the ultrasonic than the monopolar dissector. This result is in contrast with previous literature reports. Toishi et al., in their randomized study including 58 patients undergoing VATS lung lobectomy and lymph node dissection, reported significantly higher postoperative drainage volume when blunt dissection, manual ligature and/or electrocautery were preferred to vessels-sealing devices (Harmonic ACE Plus®, LigaSure™ and Enseal®) (613 ± 320 vs 437 ± 213 ml at 48 h; *p*-value = 0.0358) [5]. Similar results were reported by Yoshida et al. comparing conventional tissue dissection and vascular ligation to the use of LigaSure™ (postoperative drainage at 72 h: 705.3 ± 339.3 vs 533.8 ± 264.8 ml; *p*-value< 0.05) [4]. In our study, the concurrence of other variables may explain the increased postoperative pleural effusion production with the Harmonic ACE Plus®. In fact, the multivariable analysis identified lower lung lobectomies and increased surgery duration, but not energy device as risk factors for increased pleural effusion volume at 48 h after surgery. Moreover, after matching patients by gender, site of resection and surgeon, these differences were annulled.

Regarding chest tube duration, no differences were detected between electric hook and Harmonic ACE Plus® in our study, even when energy device variable was adjusted for other possible risk factors for late chest tube removal at multivariable analysis. Thus, beside the two cases of chylothorax in monopolar electrocautery group and the larger amount of postoperative pleural drainage in Harmonic ACE Plus® group, the use of one or the other instrument revealed a similar impact on postoperative chest tube duration. These findings are supported by the report of Schuchert et al., who did not observe any differences in terms of postoperative outcomes when Ligasure™ was used in 211 open and VATS anatomical lung resections [9]. No difference in chest tube removal timing has also been reported by Martucci et al. [10]. Conversely, Toishi et al. and Yoshida et al. reported earlier chest tube removal when vessel-sealing devices were used [4, 5]. These differences could also be explained by the fact that vessel-sealing devices were not the same in the mentioned studies [4, 5, 9, 10]. Data about postoperative prolonged air-leak, a relevant factor of delayed chest tube removal, were not reported [4, 5].

Surgical procedures lasted longer in the Harmonic ACE Plus® group of our series, while previous studies did not observe any differences in the length of surgery using vessel-sealing instruments [4, 5]. Considering these observations and our long-term favorable experience with Harmonic ACE Plus®, longer duration of surgery in the ultrasonic dissector group was probably not due to the device itself but to the higher (almost double) proportion of lower lung lobectomies performed using this device type (*p*-value = 0.005); no difference of surgery duration was detected in the matched cohort.

Finally, in terms of costs, Harmonic ACE Plus® is not reusable and is more expensive than the electric hook, which is also reusable; cost is about fourfold (~ 130 vs 500 Euros) in our institution. Thus, in the absence of documented Harmonic ACE Plus® greater impact on the surgical procedure and on postoperative short-term outcomes, electric hook may be preferred with the aim of saving direct cost of the energy device. However, ultrasonic device sealing ability could reduce the use of endoscopic staplers and ligating clips, that are also costly, and may impact on overall surgical costs [9, 14, 16, 18, 20].

This study has several limitations. First, it is an observational study with the choice of electric hook or Harmonic ACE Plus® left to surgeon’s preference. Nevertheless, we performed multivariable analysis and propensity score matching analysis to partly overcome the lack of randomization. Second, we did not analyze the Harmonic ACE Plus® performance in sealing small vessels up to 5 mm, because surgeons in our team preferred to employ endoscopic staplers or clips to close vessels not suitable for coagulation. A strength of this observational study is the evaluation and comparison of electric hook and Harmonic ACE Plus® performance in the real-world daily practice of VATS major lung resections.

## Conclusions

In the present observational study the use of either surgical monopolar electric hook or ultrasonic Harmonic ACE Plus® dissector for adhesiolysis, hilar dissection and lymphadenectomy during VATS lobectomy was safe and did not influence postoperative pleural effusion production and chest tube duration. Further randomized and larger studies are needed in order to confirm our results.

## Data Availability

The datasets used and analyzed during the current study are available from the corresponding author on reasonable request.

## Abbreviations

BMI: Body mass index
CI: Confidence interval
COPD: Chronic obstructive pulmonary disease
FEV_1_: Forced expiratory volume in 1 s
HR: Hazard ratio
IQR: Interquartile range
VATS: Video-assisted thoracoscopic surgery
